# Illegitimate work tasks: an investigation of psychometric properties of the Swedish version of the BITS instrument and its suitability in human versus ‘non-human’ service occupations

**DOI:** 10.1186/s12889-024-19393-x

**Published:** 2024-07-18

**Authors:** Johanna Stengård, Constanze Leineweber, Hanne Berthelsen

**Affiliations:** 1 Stress Research Institute, Department of Psychology, Stockholm University, Stockholm, SE-106 91 Sweden; 2 Center for Work Life and Evaluation Studies (CTA), Faculty of Odontology, Malmö University, Malmö, SE-205 06 Sweden

**Keywords:** Illegitimate tasks, BITS, Swedish version of BITS, Psychometric properties, Dimensionality, Measurement invariance

## Abstract

**Background:**

Illegitimate tasks, i.e. working tasks that are perceived as unnecessary or unreasonable, are commonly measured by the Bern Illegitimate Tasks Scale (BITS). Despite a growing research attention paid to illegitimate tasks, the Swedish version of BITS needs yet to be properly evaluated. Moreover, previous multiorganizational studies in this field have taken for granted, without really testing it, that the instrument functions invariantly in different contexts; a prerequisite for meaningful comparisons.

**Methods:**

Two occupational groups that differs hugely—966 human services workers (teachers and registered nurses) and 750 non-’human service’ workers (construction and IT-workers) were targeted utilizing questionnaires data collected 2018 within the Swedish Longitudinal Occupational Survey of Health (SLOSH) study. Psychometric properties, i.e., reliability, dimensionality, and measurement invariance between the occupations were examined using confirmatory factor analyses and structural equation models. Also, the associations between the two dimensions of illegitimate tasks and job satisfaction and emotional exhaustion, respectively, were tested.

**Results:**

Good reliability was supported and two- versus one-dimensionality showed better psychometric properties. Partial scalar measurement invariance was satisfactory supported with only 25% relaxed constraints of equal intercepts between groups. Examining the two subdimensions revealed different associations with emotional exhaustion, where the associations was more substantial for unreasonable tasks. Nevertheless, the findings point to potential improvements for future investigation.

**Conclusions:**

This study shows that the Swedish version of BITS is working satisfactory and allows for meaningful comparisons of occupational groups. Furthermore, construct validity of the two dimensions was corroborated.

**Supplementary Information:**

The online version contains supplementary material available at 10.1186/s12889-024-19393-x.

## Background

Work-related stressors are an important source of mental ill-health [1]. The global competitiveness of today’s labor market has emphasized efficiency and productivity, leading to new ways of working [2] and blurring previous boundaries of occupational descriptions. This change in previous professional role demarcations can lead to feelings of performing work tasks beyond what is reasonable or regarded as necessary, so called illegitimate tasks [3]. For examples, in health care, nurses have taken over tasks that previously were done by physicians while the physicians write patient records directly in electronic systems—a task which previously was handled by secretaries. Also, teachers complain having less time to focus on teaching due to increased administrative burden [4]. Taken together, this may lead to an increasing amount of illegitimate tasks; a work-related stressor that have been linked both to negative work attitudes and behaviors, but also decreased well-being [5].

Illegitimate tasks attract increasing attention in research. The concept refers to job tasks that are perceived to violate the rules and norms connected to the core role requirements of an occupation [3, 6]. Illegitimate tasks are related to, but distinct from, other well-established job stressors [7] such as job demands as described in Karasek’s demand-control model [8], work overload, and organizational injustice [9]. Some examples of bivariate correlations found in the literature are correlations between 0.34 and 0.46 with work overload and a correlation of 0.50 with organizational injustice [3].

In contrast to these well-known concepts of job stressors, threats (and boosts) to self-esteem constitute a central focus in the concept of illegitimate tasks [7]. To understand the nature and effect of illegitimate tasks, the Stress as Offense to Self (SOS) theory is commonly adopted. The SOS theory assumes that maintaining a positive self-image is a basic need, and any threat to self-esteem elicits strain [7]. According to the SOS theory illegitimate tasks may signal disrespect or threaten one’s valued professional identity, which in turn may negatively affect self-identity and self-esteem [7, 10]. Here, two different pathways are in work, namely Stress through Insufficiency (SIN) and Stress as Disrespect (SAD), where SIN refers to feelings of insufficiency if one’s own criteria for good performance or adequate behaviour are not met (because time and energy are spent on illegitimate work tasks). Conversely, SAD refers to the extent that one feels ignored, attacked, or excluded [7] due to the messages of disrespect contained in e.g., being given illegitimate work tasks.

### Psychometric properties of the BITS

Illegitimate tasks are typically measured by the Bern Illegitimate Tasks Scale (BITS) [3, 6]. Originally, the BITS comprised a total of nine items covering two different types of illegitimate tasks, namely unreasonable tasks (four items) and unnecessary tasks (five items) [6]. Unreasonable tasks refer to tasks that are perceived to fall outside of one’s job role requirements and unnecessary tasks refer to tasks that could have been avoided or performed with less effort if things were organized differently or other people made less mistakes. Later Semmer [3] removed one of the five items of the latter dimension because it related to both unreasonable and unnecessary tasks [6]. Unfortunately, this change has led to some confusion of which item to remove and consequently, researchers have used different versions of the instrument since then [11]. Despite this, the two-factor solution with the subdimensions unreasonable tasks and unnecessary tasks has repeatedly shown superior psychometric properties compared to a single-factor solution combining all items [6, 11–14]. However, although the majority of scholars acknowledge the two-dimensionality of BITS, the measure is often studied in terms of a single dimension [5]. This approach may disguise important knowledge about the implications of illegitimate tasks and, as a matter of fact, a couple of studies have reported inconsistent findings in terms of unreasonable and unnecessary tasks in terms of their relationship with other psychosocial constructs (see for example, illegitimate tasks with regard to job crafting and meaning of work [15] and illegitimate tasks and intrinsic motivation [14]).

### The validity of the BITS among different groups/occupations

In many cases, it is of great relevance to compare the extent of illegitimate tasks across different groups, for example in workplace surveys or in epidemiological studies. For this, it is a prerequisite that the instrument works invariantly across groups for the comparison to reflect true differences rather than measurement bias [16]. In a validation study of the Polish version of BITS, Basinska and Dåderman found the instrument worked invariantly for men and women [11] and a few studies have shown that it works invariantly between countries [17, 18]. However, to our best knowledge BITS comparability across different occupational groups has not yet been investigated.

As outlined above, there is some confusion in regard to the exact number and formulation of items. In the Swedish version of BITS [19] this confusion is also reflected. The present study builds on an established Swedish version (see Methods section for details), which is operationalized including the item that Semmer [3] suggested to omit instead of the item they suggested to keep in the instrument. Swedish studies differ in the number of dimensions analyzed and reported, for example, some scholars use an overall illegitimate tasks measure [20, 21], while others analyze the two subdimensions separately [19, 22].

Besides the important aspect of examining the dimensionality of the Swedish version of the BITS, associations with other psychological constructs found in the international literature should be replicated as part of validation. For example, several international studies support associations between BITS and adverse outcomes, such as burnout/emotional exhaustion [23–25] and low job satisfaction [11, 26]. Studies in the Swedish context have found corresponding associations with for example stress and mental exhaustion [19, 22, 27], but also with dissatisfaction with work performance [20].

In summary, further validation of the BITS is needed for the Swedish context regarding its dimensionality and how it works across different occupational settings, since an instrument needs to be validated in every new context it is used [28]. Importantly, measurement invariance needs to be established, to assure that psychometric properties of the BITS are transferable or generalizable across different occupational groups, meaning that a group difference in level of illegitimate tasks likely reflect a true difference between the groups.

### Aim of the current study

The purpose of the present study is to evaluate the psychometric properties of the Swedish version of the BITS in different occupational settings. More specifically we aim to examine: 1) the dimensionality and reliability of the BITS and its associations with emotional exhaustion and job satisfaction, and 2) if the BITS functions invariantly in two very different types of occupations (i.e. human service workers (teachers and registered nurses) and non-’human service’ workers (construction and IT workers)).

## Methods

### Study population

Data were drawn from the Swedish Occupational Survey of Health (SLOSH) study; a panel-survey with data collection every second year (since 2022 every year), focusing on the complex relationships between work organization, work environment, labour market participation and health. SLOSH started in 2006 with a first follow-up of participants in the Swedish Work Environment Survey (SWES) 2003. Since, later SWES waves have subsequently been added and at the time this study was conducted, SLOSH consisted of all SWES participants 2003–2011 (n = 40,877). Participants were asked to fill in a postal questionnaire in either of two versions, one for those currently in paid work and one for those permanently or temporarily outside the labour force. Analyses were restricted to those who answered the questionnaire for those in paid work in 2018 (exclusive self-employed individuals) and who had answered all BITS items. Further, in accordance with our aim, we chose four different occupational groups, based on the Swedish Standard Classification of Occupations (SSYK 2012) [29], namely registered nurses (SSYK 2220–2239), teachers (SSYK 2320, 2330 and 2341), construction workers (SSYK 7100–7233), and IT workers (SSYK 2510–2519). This resulted in a sample with 966 human service workers (registered nurses (n = 464) and teachers (elementary and upper secondary school teachers (n = 502)) and 750 non-’human service’ workers (construction (*n* = 426) and IT (*n* = 324) workers). These groups were chosen to achieve variation in relation to the nature of the work (working with subjects/objects), the typical sector (public/private) and gender composition. The mean age of the study sample was 52.5 (SD 9.6) years, about 55% were women (*n* = 938), about 89% worked more than 30 h/week (*n* = 1,510), 95% had a permanent contract and in average they had been working in their organization for 14.9 (SD 12.4) years.

### Measurements

Illegitimate tasks were measured by the Swedish version [19] of the Bern Illegitimate task Scale (BITS) [3, 6]. Four items refer to unreasonable tasks and four items to unnecessary tasks. The formulation of items is presented in the appendix (see Supplementary Table S1). In the existing Swedish version [19], the BITS item “...they would not exist (or could be done with less effort), if some other people made less mistakes?” is included (UnT4), and the BITS item “… they just exist because some people simply demand it this way?” is omitted. All items are answered on a 5-point Likert scale reaching from ‘never’ [1] to ‘very often’ [5].

Emotional exhaustion was measured by 7 items of the Emotional Exhaustion subscale of the Shirom-Melamed Burnout Scale [30, 31]. Answers were given on a Likert scale with seven options, reaching from ‘almost never’ [1] to ‘almost always’ [7]. A mean-index was constructed. Job satisfaction was measured by a single item, “Roughly, how satisfied are you with your work?” that is answered on an 8-points Likert scale reaching from 1= ‘very unsatisfied’ to 8 = ‘very satisfied’.

### Analytical strategy

For data cleaning, variable creation and descriptive statistics Stata/SE 17.0 [32] was used and for more complex analyses Mplus 8.8 [33] was used.

#### Confirmatory factor analyses

The factor structure of the BITS items was examined by confirmatory factor analyses (CFA) with the robust maximum likelihood (MLM) estimator, which is robust to non-normality [34]. CFA was performed for the total study sample and for each of the four occupational groups separately. The following model fit indices were utilized to examine fit between the proposed model and the sample data: The comparative fit index (CFI), the Tucker-Lewis index (TLI), the standardized root mean squared of residuals (SRMR), and the root mean square error of approximation (RMSEA). Values of CFI and TLI ≥ 0.95, RMSEA ≤ 0.05, and SRMR ≤ 0.05 indicate good fit, whereas values of CFI and TLI > 0.90 and RMSEA < 0.08 indicate acceptable fit [34]. One and two-factor models (with and without the originally omitted item) were tested and compared.

#### Construct validity

Following the recommendations for structural equation models (SEM) by Cheung [28], reliability and construct validity were assessed by the means of multiple criteria. *Construct Reliability* was assessed with McDonald’s omega, which, unlike Cronbach’s alpha, does not assume equal factor loading across indicators [35], using the widely applied cut-off value of > 0.7 [36]. *Convergent validity*, in addition to evaluating model fit in CFA model, was assessed by examining average variance extracted (AVE) > 0.5 [37] for each latent variable. *Discriminant validity* was assessed by means of no cross-loaded indicators [38] and by ensuring that the upper limit of the confidence interval of the correlation between two factors (CI_CFA_) < 0.8 [39]. Also, to further strengthen construct validity, associations between the two BITS factors and the two outcomes emotional exhaustion and job satisfaction, were assessed in SEM with the estimator MLM. These analyses were run for the total study sample and in stratified analyses by human service and non-’human service’ workers, using multigroup SEM analysis.

#### Measurement invariance

To investigate whether the BITS was understood similarly and results could be compared across different occupational settings, measurement invariance was tested by comparing a series of nested multigroup confirmatory factor analyses (configural, metric and scalar invariance models). Configural invariance implies that the same set of items loads on the same factors across groups, metric invariance implies that the magnitude of these item-loadings (factor loadings) are similar across groups, and finally scalar invariance implies that the item intercepts are similar across the groups. Before, merging the selected human service occupations together and non-’human service’ occupations together, we tested measurement invariance within human service occupations (registered nurses vs. teachers) and measurement invariance within non-’human service’ occupations (construction vs. IT workers).

To evaluate measurement invariance, we followed the recommendations by Chen [40] promoting the changes in fit indices between two nested models over using the chi-square difference testing, where the latter in large samples is sensitive even to minor parameter changes. More specifically, a change of ≥ 0.010 in CFI, accompanied by a change of ≥ 0.015 in RMSEA or a change of ≥ 0.030 in SRMR would indicate non-invariance for metric invariance testing, whereas for scalar invariance testing the allowed change of SRMR is a bit more conservative, i.e. ≥ 0.010 [40].

## Results

### Sample descriptive

Table 1 shows sample characteristics for both the total sample and stratified by the four occupations (i.e., registered nurses, teachers, construction workers, and IT workers). As it was implied and expected, the gender distribution differed heavily between the four occupations. A majority of women were found in the human service category (90.7% women among registered nurses and 75.3% among teachers) whereas almost the opposite distribution was found in the non-’human service’ category (5.9% women among construction and 35.2% among IT workers). The two main occupational groups under scrutiny differed also in other anticipated ways: With regard to educational level, the category covering human service occupations were well-educated (99.8% nurses and 96.2% teachers with higher education), while the non-’human service’ category had a more diverse distribution in regard to educational level; IT workers were well-educated (80.8%) while few construction workers had gained higher educational level (7.5%). Further, a higher amount of the human service workers compared to non-’human service’ workers worked in the public sector. In other aspects—i.e., working time, employment type, management positions, mean age, and tenure—the two occupational categories did not differ substantially. For detailed descriptive statistics of the eight BITS items with respect to the total study sample as well as stratified by human service occupations (nurses and teachers) and non-’human service’ occupations (construction and IT workers), see Supplementary Table S2.

**Table 1:** Descriptive of the samples

	Nurses (*n* = 464)	Teachers (*n* = 502)	Construction workers (*n* = 426)	IT workers (*n* = 324)	Total Cases (*n* = 1,716)*
	*n* (%)	*n* (%)	*n* (%)	*n* (%)	*n* (%)
**Sex**					
Men	43 (9.3)	124 (24.7)	401 (94.1)	210 (64.8)	778 (45.3)
Women	421 (90.7)	378 (75.3)	25 (5.9)	114 (35.2)	938 (54.7)
**Education**					
Secondary school	1 (0.2)	19 (3.8)	394 (92.5)	62 (19.3)	476 (27.8)
Higher education	463 (99.8)	482 (96.2)	32 (7.5)	260 (80.8)	1,237 (72.2)
**Working at least 30 h/week**					
< 30 h/week	90 (19.6)	52 (10.5)	22 (5.3)	19 (6.0)	183 (10.8)
≥30 h/week	370 (80.4)	443 (89.5)	397 (94.8)	300 (94.0)	1,510 (89.2)
**Employment type**					
temporary	29 (6.3)	33 (6.7)	10 (2.4)	4 (1.3)	76 (4.5)
permanent	430 (93.7)	460 (93.3)	404 (97.6)	316 (98.8)	1,610 (95.5)
**Public sector**					
Private	58 (12.7)	43 (8.9)	387 (95.8)	245 (80.1)	733 (44.5)
Public	399 (87.3)	438 (91.1)	17 (4.2)	61 (19.9)	915 (55.5)
**Patient/student responsibility**					
No	86 (18.6)	19 (3.8)	414 (98.1)	324 (100.0)	843 (49.4)
Yes, < 50% working time	63 (13.6)	24 (4.8)	5 (1.2)	0 (0)	92 (5.4)
Yes, ≥ 50% working time	313 (67.8)	456 (91.4)	3 (0.7)	0 (0)	772 (45.2)
**Manager/ supervisor**					
no	256 (56.6)	395 (84.8)	328 (81.4)	195 (61.5)	1174 (71.7)
yes	196 (43.4)	71 (15.2)	75 (18.6)	122 (38.5)	464 (28.3)
**Age** mean (st.dev.)	52.8 (10.1)	53.6 (9.5)	53.6 (9.3)	49.1 (8.7)	52.5 (9.6)
**Tenure** (in years)	14.7 (13.2)	16.6 (12.6)	15.9 (12.6)	11.1 (9.8)	14.9 (12.4)

### Factorial structure

**Fig. 1:**
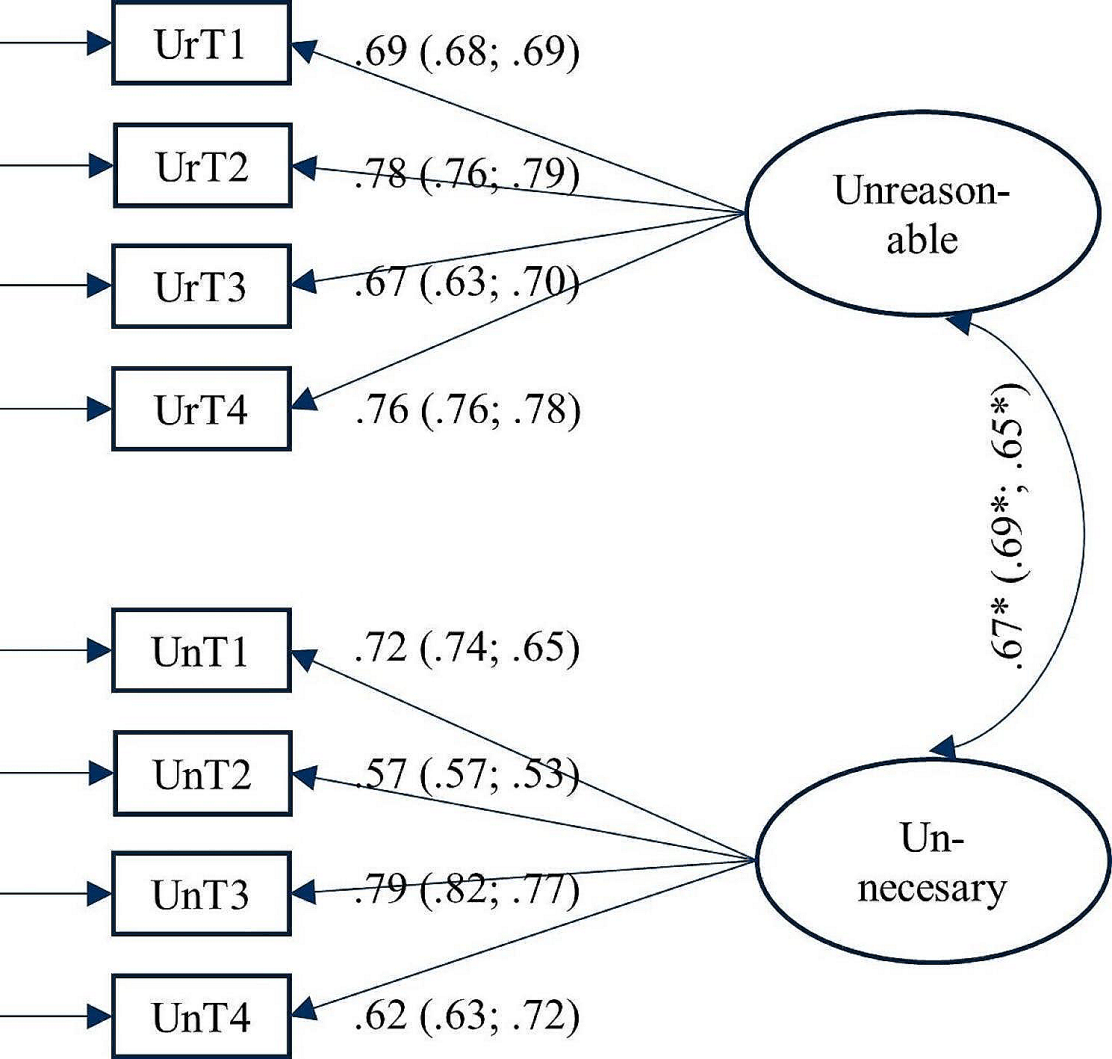
The final Confirmatory factor analysis (CFA) solution with the two factors unreasonable and unnecessary tasks. Standardized item factor loadings are presented for the total study sample followed in parentheses by stratified (by multigroup analysis) factor loadings for the two occupational categories (registered nurses and teachers; construction and IT workers) Note. Total study sample, *N =* 1,716 individuals; Human service workers sample, *n =* 966 individuals; Non-‘human service’ workers sample, *n =* 750 individuals No modification adding error-covariances between items included UrT is unreasonable tasks; UnT is unnecessary tasks; * = *p* < .05

Confirmatory factor analyses of the BITS supported a latent construct with a two-factor structure—unreasonable and unnecessary tasks—over a one-factor structure. This means that, as expected, four BITS items loaded on unreasonable tasks and the other four on unnecessary tasks. No item loaded on both factors and consequently no modifications were made. The two-factor model showed satisfactory fit indices: χ2(df) = 182.82 [19] with estimator MLM, CFI = 0.959, RMSEA = 0.071 (90% CI 0.062–0.080) and SRMR = 0.030 for the total study sample as well as for human service and non-’human service’ subsamples in (multigroup) stratified analysis (Table 2). Figure 1 shows standardized factor loadings for the two-factor solutions. For better comparability with international literature, we also run a model excluding the item UnT4 (see Table S1 “...if other people made less mistakes”). Also, this model showed satisfactory fit indices: χ2(df) = 63.4 [13], CFI = 0.985, RMSEA = 0.048 (90% CI 0.036–0.060) and SRMR = 0.022 (Supplementary Table S3; Figure S1) and the bivariate correlation between the 4-item scale and the 3-item scale was very high (*r* = .96). As a consequence, we also performed all tests excluding the item UnT4 entirely, which can be find in supplementary tables.

**Table 2:** Confirmatory factor analyses BITS. Fit indices

Model	χ2(df)*	CFI	RMSEA	SRMR
1-factor solution, total sample (*N* = 1,716)	686.2 [20]	0.832	0.139 (0.130-0.148)	0.070
2-factors solution, total sample (*N* = 1,716)	182.8 [19]	0.959	0.071 (0.062-0.080)	0.030
2-factors solution, human service workers (*n* = 966)	98.7 [19]	0.964	0.066 (0.053-0.079)	0.030
2-factors solution, non-‘human service’ workers (*n* = 750)	99.8 [19]	0.954	0.075 (0.061-0.090)	0.039

* with estimator MLM

### Reliability and construct validity

McDonald’s omega was acceptable (greater than 0.7) for all latent constructs (the two BITS factors and emotional exhaustion) both in the total study sample and in in the two sub-samples (Table 3). With regard to convergent validity, average variance extracted (AVE) was greater than 0.5 for each latent construct—in the total sample and in stratified samples—except for unnecessary tasks, which was slightly under the suggested cut-off. Discriminant validity was supported as: (1) no cross-loaded indicators were found, neither in explorative factor analysis (EFA) nor by modification indices derived from Mplus output of the CFA models and (2) the correlation between the two BITS factors was 0.67 (95%CI: 0.63–0.72), thus the upper limit for the 95% confidence interval was lower than 0.8 in accordance with Rönkkö and Cho [39] Similar results were found for analyses excluding the item UnT4, except for a slightly better AVE for unnecessary tasks (AVE = 0.49 for the total study sample).

**Table 3:** Descriptive statistics of the study variables. Total study sample and per occupation

	Total study sample		Human service		Non-‘human service’		Bivariate correlations^c^ (total study sample)			
	ω	AVE	ω	AVE	ω	AVE	Emotional exhaustion	Job satisfaction	Unreasonable tasks	Unnecessary tasks
Emotional exhaustion^a^	.921	.630	.932	.668	.903	.577	-			
Job satisfaction	NA	NA	NA	NA	NA	NA	− .447	-		
Unreasonable tasks	.817	.529	.802	.507	.829	.549	.381	− .355	-	
Unnecessary tasks	.769	.458	.782	.476	.766	.456	.305	− .306	.534	-
Unnecessary tasks (3 items)^b^	.742	.490	.757	.510	.707	.446	.279	− .289	.514	.960

Note. ω is McDonald’s Omega; AVE is average variance extracted

^a^ N = 1,683

^b^ excluding the item UnT4 (“...if other people made less mistakes”)

^c^ based on mean values for each variable

### Measurement invariance (multigroup CFA results)

First, we examined whether it was justified to pool the two human service occupation samples as well as the two non-’human service’ occupation samples together. With respect to registered nurses and teachers, metric invariance was supported, but not full scalar invariance (Table 4). However, considering model modification indices (derived from Mplus output) we released the constraints of equal intercept of the item UnT4 (“…if other people made less mistakes”) of the unnecessary tasks scale. Doing so, partial scalar invariance was attained. Thus, a pooling of the samples for registered nurses and teachers into one category of human service workers was supported. The measurement invariance analyses for construction and IT workers reached the highest examined level of measurement invariance, i.e., scalar invariance (Table 5). However, also for these two groups the fit indices improved substantially when relaxing the restriction of the same item intercept as for human-service workers. Consequently, we pooled the samples for construction and IT workers into non-’human service’ workers.

**Table 4:** Fit indices for measurement invariance testing in human service workers (nurses vs. teachers)

Model	χ2 (df)	CFI	RMSEA (90% CI)	SRMR	Model comp	Δχ2 (Δdf)^b^	ΔCFI	ΔRMSEA	ΔSRMR
M1: Configural Invariance	119.1 (38)	0.964	0.066 (0.053-0.080)	0.035	--	--	--	--	--
M2: Metric Invariance	130.1 (44)	0.962	0.064 (0.051-0.076)	0.040	M2 vs. M1	11.1 (6)	0.002	0.002	0.005
M3: Scalar Invariance	186.4 (50)	0.940	0.075 (0.064-0.087)	0.050	M3 vs. M2	61.5*** (6)	0.022	0.011	0.010
M3a: Partial Scalar Invariance	154.8 (49)	0.953	0.067 (0.055-0.079)	0.044	M3a vs. M2		0.009	0.003	0.004

Note. *N* = 966; Nurses, *n* = 464; Teachers; *n* = 502

*** *p* < .001

^a^ the constraints of equal intercepts for one item (UnT4: “…if other people made less mistakes”) of the unnecessary tasks’ dimension was relaxed

^b^ with MLM correction

**Table 5:** Fit indices for measurement invariance testing in Non-‘human service’ workers (Construction vs. IT workers)

Model	χ2 (df)	CFI	RMSEA (90% CI)	SRMR	Model comp	Δχ2 (Δdf)^b^	ΔCFI	ΔRMSEA	ΔSRMR
M1: Configural Invariance	119.5 (38)	0.955	0.076 (0.061-0.091)	0.046	--	--	--	--	--
M2: Metric Invariance	128.3 (44)	0.953	0.071 (0.057-0.086)	0.050	M2 vs. M1	7.9 (6)	0.002	0.005	0.004
M3: Scalar Invariance	177.3 (50)	0.929	0.082 (0.069-0.096)	0.058	M3 vs. M2	54.5*** (6)	0.024	0.011	0.008
*M3a: Partial Scalar Invariance*	*155.5 (49)*	*0.941*	*0.076 (0.063-0.090)*	*0.054*	*M3a vs. M2*		*0.005*	*0.005*	*0.004*

Note. *N* = 750; Construction workers, *n* = 324; IT workers, *n* = 426

*** *p* < .001

^a^ the constraints of equal intercepts for one item (UnT4: “…if other people made less mistakes”) of the unnecessary tasks’ dimension was relaxed

^b^ with MLM correction

In a final step, we tested measurement invariance comparing the human service with the non-’human service’ occupation samples. These tests reached metric invariance, but not full scalar invariance. Modification indices indicated that for attaining partial scalar invariance, in addition to relaxing the restriction for the intercept of the previous discussed unnecessary tasks item, also one item of the unreasonable tasks factor (UrT3: “…put you into an awkward position”) had to be relaxed (Table 6). In supplementary analyses, excluding item UnT4 (“…if other people made less mistakes”) completely, full scalar invariance was supported in analyses within human service occupations (registered nurses vs. teachers) and within non-’human service’ occupations (construction vs. IT workers). Between human service occupations and non-’human service’ occupations, metric invariance was supported and partial scalar could be achieved when releasing the constraint of equal intercept for the item UrT3 (“…put you into an awkward position”) (Supplementary Table S4-S6).

**Table 6:** Fit indices for measurement invariance testing between human service workers and non-‘human service’ workers

Model	χ2 (df)	CFI	RMSEA (90% CI)	SRMR	Model comp	Δχ2 (Δdf)^b^	ΔCFI	ΔRMSEA	ΔSRMR
M1: Configural Invariance	198.5 (38)	0.960	0.070 (0.061–0.080)	0.034	--	--	--	--	--
M2: Metric Invariance	229.5 (44)	0.954	0.070 (0.061–0.079)	0.043	M2 vs. M1	30.9*** (6)	0.006	0.000	0.009
M3: Scalar Invariance	439.5 (50)	0.903	0.095 (0.087–0.104)	0.062	M3 vs. M2	239.2*** (6)	0.051	0.025	0.019
M3a: Partial Scalar Invariance	259.4 (48)	0.947	0.072 (0.063–0.080)	0.045	M3a vs. M2		0.007	0.002	0.002

Note. *N* = 1,716; Human service workers (nurses and teachers), *n* = 966; Non-‘human service’ workers (construction and IT workers), *n* = 750

*** *p* < .001

^a^ the constraints of equal intercepts for one item (UnT4: “…if other people made less mistakes”) of the unnecessary tasks’ dimension and one item (UrT3: “…put you into an awkward position”) of the unreasonable tasks’ dimension were relaxed

^b^ with MLM correction

### Associations between BITS and emotional exhaustion and job satisfaction

**Fig. 2:**
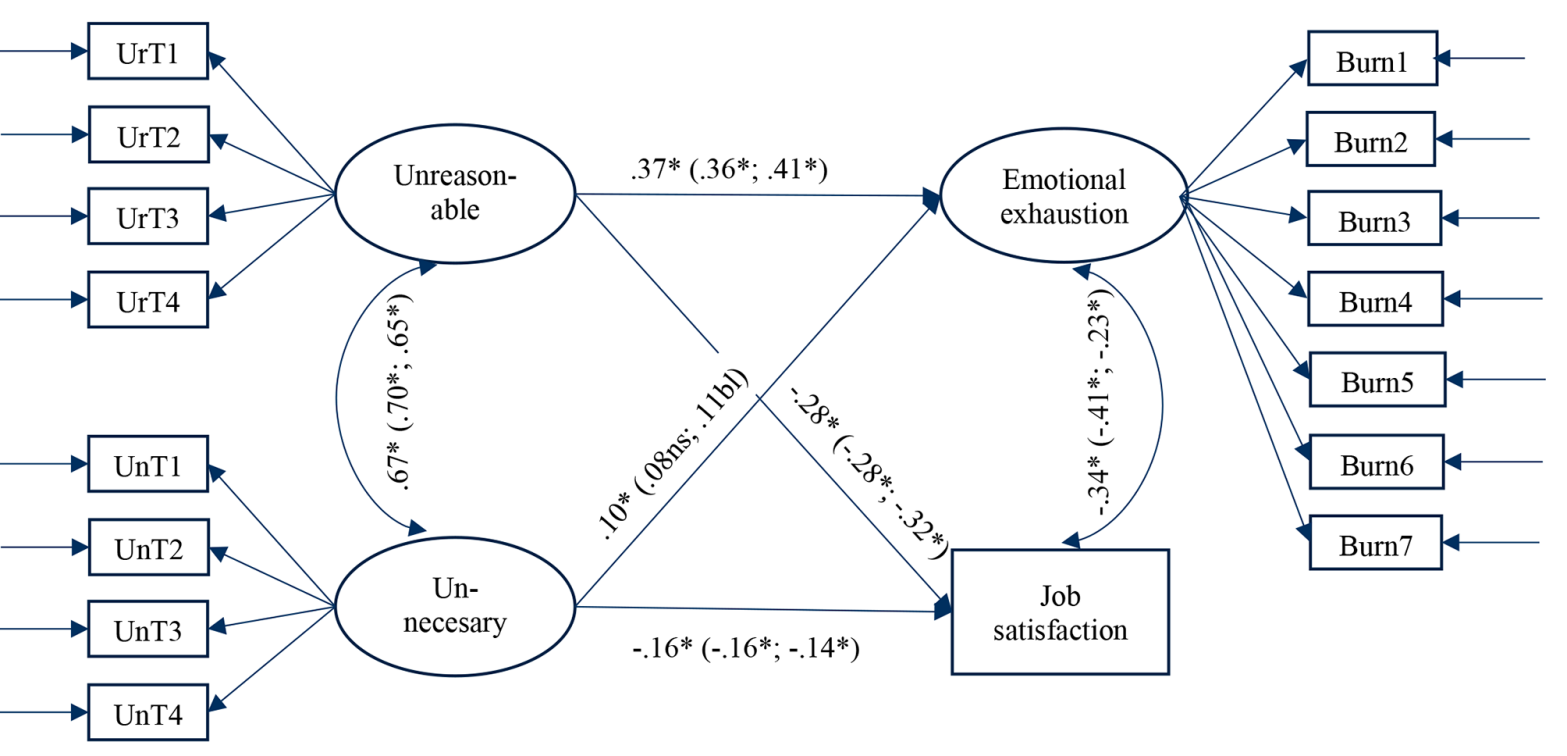
Standardized associations: All groups (in parentheses; results from multigroup analyses: HSW (registered nurses & teachers); non-HSW (construction and IT workers)). No modification adding error-covariances between items was used. Fit indices for total study sample χ^2^(df) = 472.30(99), CFI = 0.966, RMSEA = 0.048 (90% CI 0.043–0.052) and SRMR = 0.029); for (partial scalar) multigroup analysis χ^2^(df) = 692.38(220), CFI = 0.958, RMSEA = 0.051 (90% CI 0.046–0.055) and SRMR = 0.043) UrT is unreasonable tasks; UnT is unnecessary tasks; * = *p* < .05; ns = non-significance; bl = borderline significance

Good fit was achieved for a SEM model (χ^2^(df) = 472.30(99), CFI = 0.966, RMSEA = 0.048 (90% CI 0.043–0.052), and SRMR = 0.029) including the total study sample, where the expected associations between both BITS factors, respectively, and emotional exhaustion and job satisfaction were supported (Fig. 2). The association between unreasonable tasks and emotional exhaustion was more substantial (β = 0.371, *p < .*001) than the association between unnecessary tasks and emotional exhaustion (β = 0.100, *p = .*017) (Wald’s test 10.25 [1], *p = .*0014, from unstandardized analyses). Also, associations between unreasonable tasks and job satisfaction (β= -0.278, *p < .*001) and between unnecessary tasks and job satisfaction (β= -0.164; *p < .*001) were supported. In (partial scalar invariance) multigroup SEM analysis (χ^2^(df) = 692.38(220), CFI = 0.958, RMSEA = 0.051 (90% CI 0.046–0.055), and SRMR = 0.043) comparing human-service vs. non-‘human service’, all associations remained significant for both groups, except the association between unnecessary tasks and emotional exhaustion which turned non-significant (*p = .*156) for human-service workers and borderline significant (*p = .*080) for non-‘human service’ workers. Similar results were found for analyses excluding the item UnT4 (“.if other people made less mistakes”) (Figure S2).

## Discussion

To evaluate the psychometric properties of the Swedish version of the BITS and its validity in different occupational settings in a Swedish work context, the present study focused on occupations in two very different settings/sectors: 1) human service workers (i.e., registered nurses and teachers) working in typically women-dominated sectors and 2) non-’human service’ workers (i.e., construction and IT workers) working in typically men-dominated sectors. As anticipated the two-factor solution—consisting of the two subdimensions unnecessary tasks and unreasonable tasks—proved good reliability and substantially better psychometric properties than a single-factor solution. Furthermore, the instrument was found to work satisfactory in both occupational domains and expected associations with emotional exhaustion and job satisfaction were supported.

### Dimensionality and associations with other constructs

In accordance with previous international studies [6, 11–14], the two-factor solution of the Swedish version of BITS showed substantially better psychometric properties than a single-factor solution. Moreover, both subdimensions—unreasonable and unnecessary tasks—showed good reliability. Accordingly, separating the two subdimensions of illegitimate tasks is preferable from a psychometrical perspective, besides from a theoretical point of view: unnecessary tasks refer to tasks which justification of existence are questioned by the employee and may be considered simply as a waste of time, whereas regarding unreasonable tasks “it is not the task as such that is illegitimate; it is its social meaning in relation to one’s work role” [7](p.275), thus probably being a stronger threat to one’s self-esteem.

The effect sizes of unreasonable and unnecessary tasks on job satisfaction found in the present study were about the same as found in previous studies [11, 26]. With regard to associations between illegitimate tasks and emotional exhaustion, previous studies, sometimes measuring one single or two separate dimensions, show a large range of effect sizes [23–25]. In the present study the association between unreasonable tasks and emotional exhaustion was more substantial than the one between unnecessary tasks and emotional exhaustion, where the latter when stratified by human-service vs. non-‘human service’ workers turned non-significant. This is in line with the SOS-theory, suggesting that unreasonable tasks, which to a greater extent may signal that the employee is not valued, may be more devastating to the person than unnecessary tasks [6]. Another study supporting this notion found that hostile attribution bias moderated the association between particularly unreasonable tasks and negative emotions, such that individuals with stronger hostile attribution bias were more affected by high unreasonable tasks [41]. This further strengthens—besides the better psychometric properties of the two-factor solution compared to the one-factor solution—the rationale to study the two subdimensions of BITS separately in relation to other psychological constructs.

### Measurement invariance of BITS between occupational sectors

With respect to registered nurses and teachers, metric measurement invariance was shown, indicating that each item contributed to the subdimensions to a similar degree across the two groups. However, *full* scalar invariance was not attained, which may indicate measurement bias such that respondents in one group compared to the other group systematically rate a certain item higher or lower (in contrary to the other items of the dimension), disrupting the pattern. Similar results were found when comparing human service workers with non-’human service’ workers. However, *partial* scalar invariance was achieved by partially relaxing the requirement that all item intercepts be equal between compared groups. More specifically, the constraints of equal intercepts for one item (UnT4: “…if other people made less mistakes”) of the unnecessary tasks’ dimension needed to be relaxed when testing registered nurses versus teachers, and in subsequent comparisons between human service and non-’human service’ workers, additionally the constraints of equal intercepts for one of the items (UrT3: “…put you into an awkward position”) of unreasonable tasks’ dimension had to be relaxed.

The former finding is in line with the conclusion by Semmer [3], who decided to exclude the item UnT4 (“...if other people made less mistakes”) entirely from the scale, due to problems with double loadings. In our data, the problem of double loading was not an issue; however, this particular item was the only item where the non-‘human service’ workers in general reported a higher value than the human-service workers. Also, AVE for the unnecessary tasks’ subdimension was slightly below the recommended threshold of 0.50, which means that the construct could explain less than 50% of the variance in the items. Still, some scholar claims that this threshold for AVE is too restrictive and that a good construct reliability is enough for proving convergent validity [42, 43]. Excluding this particular item entirely, slightly improved the AVE approaching the threshold for the total sample and exceeded it for the human services sample. However, for the non-’human service’ sample it was the opposite, removing the item slightly decreased the AVE. In future studies on the Swedish version of the BITS, we recommend to decide based upon psychometric properties for the particular study sample whether to keep the item UnT4 (“...if other people made less mistakes”) or not. For future data collection, also the missed item from the original scale (“…others want it that way”), should be included for future psychometric properties testing.

Regarding the item UrT3 (“…puts you in awkward situations” (in the Swedish version translated to ‘obehagliga’, which in English corresponds to ‘unpleasant’)), part of the unreasonable subdimension, one could argue that it has a slightly different meaning than the other items of the subdimension. Are unpleasant situations (or for that matter awkward situations) during a work day per se illegitimate and should always be done by others? For example, a physician informing a patient about a severe diagnosis or a relative about the death of a loved one, or for that matter a project manager having to inform a subordinate that he/she has implemented substandard software code or construction work. According to Semmer and coauthors “an illegitimate task may put people in an awkward position, as when they have to communicate a negative decision that a supervisor has made but does not want to communicate it him- or herself” [3] (p.24) and “it puts the employee into an awkward position (like having to take responsibility for somebody else’s mistake when interacting with customers or delivering negative feedback to a colleague)” [6] (p.43). However, the item does not clarify whether such a difficult task is part of one’s professional role or not. Perhaps this should be clarified in future versions. Inspecting the original German scale, this particular item had a slightly different meaning (“Gibt es Tätigkeiten, von denen Sie der Meinung sind, dass man Sie in eine unmögliche Situation gebracht hat” in German [6]), where a more appropriate translation of ‘unmögliche Situation’ into Swedish perhaps may be ‘omöjlig’ (‘impossible’ in English). Another possible objection to the wording ‘unpleasant’ may be that it could be interpreted targeting physical working conditions. Therefore, we propose that scholars in the future add a new question closer to the original meaning of the item and once again test the measurement properties of the Swedish version of the scale.

### Strengths and limitations

Strengths of this study is the approximately representative sample of the Swedish working population and that with rigorous testing using high standard methods, we examined psychometric properties of BITS and in previous studies established cross-sectional associations between the subdimensions of illegitimate tasks and other psychological constructs. Although sufficient for the purposes of this study, future studies should study these relationships longitudinally, and thus also enabling the investigation of measurement invariance across time.

The problem of not attaining (full) scalar invariance, could indicate that a found group-difference may not reflect a true difference between the groups, since one of the groups may evaluate their levels of one or more particular item(s) higher even though the groups in fact are exposed to similar levels of the item(s). However, in the present study, we attained partial scalar invariance by the relaxation of only one out of four item intercepts for each subdimension, which we consider satisfactory in light of guidelines limiting non-invariant items of a certain construct to less than 50% [44]. To further justify relaxation of certain item intercept constraints, according to Chen [45] one should compare the original full scalar model with the relaxed one (partial scalar model), evaluating that similar substantive conclusions of interest could be drawn with regard to mean-value differences and associations with other constructs. This was the case—comparing the two SEM models (full vs. partial scalar models) in terms of mean values of illegitimate tasks’ subdimensions and their respective associations with emotional exhaustion and job satisfaction—and hence, we conclude that the level of measurement invariance was satisfactory. Also, the two subdimensions of the Swedish version have previously shown good reliability [19, 27]. As a matter of fact, a recent Pakistani study [26] ended up with the very same set of eight items as constitutes the Swedish version [19] after the completion of CFA with the nine original items. They found that the very same item, that is not part of the Swedish version, had a factor loading with insufficient magnitude and as a consequence was excluded in their study [26].

To sum up, our findings support that the Swedish version of the BITS works adequate in two very different occupation settings/sectors with very different work environment and demographic background.

## Conclusions

The findings of the present study suggest that the Swedish version of the BITS can be used in several occupational settings, both human and non-’human service’ occupations, and be used to make meaningful comparisons. Also, the support for the two- over the one-factor solution and the found difference in effect size for the associations between the two subdimensions and burnout is promising. Further investigation of the two subdimensions’ separate relations to health outcomes and well-being can be used in future studies to reveal which aspects of illegitimate tasks are more important for certain outcomes.

### Electronic supplementary material

Below is the link to the electronic supplementary material.

Supplementary Material 1

## Data Availability

The datasets generated and/or analysed during the current study are not publicly available due to restrictions from the ethical review board and considering that sensitive personal data are handled, but are available on reasonable request. Access to the data may be provided to other researchers in line with Swedish law and after consultation with the Stockholm University legal department. Requests for data, stored at the Stress Research Institute, Department of Psychology, should be sent to registrator@su.se with reference to ‘Illegitimate tasks 2022-13’ or directly to the corresponding author.

## Abbreviations

AVE: average variance extracted
BITS: Bern illegitimate tasks scale
CFA: confirmatory factor analyses
CFI: comparative fit index
CI: confidence interval
EFA: explorative factor analysis
RMSEA: root mean square error of approximation
SAD: stress as disrespect
SEM: structural equation model
SIN: stress through insufficiency
SLOSH: Swedish longitudinal occupational survey of health
SOS: stress as offense to self
SRMR: standardized root mean squared of residuals
SSYK: Swedish standard classification of occupations
SWES: Swedish work environment survey
TLI: Tucker-Lewis index
UrT: unreasonable tasks
UnT: unnecessary tasks
