# Supplementary material for: Illegitimate work tasks: an investigation of psychometric properties of the Swedish version of the BITS instrument and its suitability in human versus ‘non-human’ service occupations
Source: BMC Public Health. 2024 Jul 18;24:1935. doi: 10.1186/s12889-024-19393-x (PMC11264809; doi:10.1186/s12889-024-19393-x)
Supplement: Supplementary file 1 — Supplementary Material 1 [file 12889_2024_19393_MOESM1_ESM.docx]

**Table S1.** BITS items. English version^a^ /*Swedish version*^b^.

| Unreasonable tasks | Do you have work tasks to take care of, which you believe … /  *Måste du utföra arbetsuppgifter som du menar ...* |
| --- | --- |
| UrT1 | …should be done by someone else? / *...borde göras av någon annan?* |
| UrT2 | …are going too far, which should not be expected from you? */ ...kräver mer av dig än vad som är rimligt?* |
| UrT3 | …put you into an awkward position? / *... försätter dig i obehagliga situationer?* |
| UrT4 | …are unfair that you have to deal with them? / *...att det är orättvist att de hamnat hos dig?* |
| **Unnecesary tasks** | **Do you have work tasks to take care of, which keep you wondering if … /**  ***Har du arbetsuppgifter som du undrar över om de ...*** |
| UnT1 | …they have to be done at all? / *...egentligen alls behöver göras?* |
| UnT2 | …they make sense at all? / *...är vettiga och meningsfulla?* |
| UnT3 | …they would not exist (or could be done with less effort), if things were organized differently? / *...alls borde finnas (eller kunde göras med mindre ansträngning) om saker och ting var annorlunda organiserade?* |
| UnT4^c^ | …they would not exist (or could be done with less effort), if other people made less mistakes? / *...alls borde finnas (eller kunde göras med mindre ansträngning) om vissa andra gjorde färre misstag?* |
| Note.  ^a^ Jacobshagen, 2006; Semmer et al., 2010. ^b^ Aronsson & Mellner, 2016 (slightly adapted).  ^c^ In the latest English version of the scale, this particular item is excluded and another item is included. | |

**Table S2.** Descriptive statistics of the BITS items

|  | All;  N_tot_ = 1,716 | | | Human service work  (teachers and nurses);  N = 966 | | | Non-‘human service’ work  (construction and IT workers); N = 750 | | |
| --- | --- | --- | --- | --- | --- | --- | --- | --- | --- |
| Item | Mean (SD) | Skewness | Kurtosis | Mean (SD) | Skewness | Kurtosis | Mean (SD) | Skewness | Kurtosis |
| UrT1 | 2.89 (0.95) | 0.130 | -0.067 | 3.02 (1.01) | 0.027 | -0.227 | 2.73 (0.83) | 0.101 | 0.069 |
| UrT2 | 2.61 (0.92) | 0.412 | 0.125 | 2.75 (0.97) | 0.316 | -0.090 | 2.44 (0.82) | 0.414 | 0.361 |
| UrT3 | 2.38 (0.85) | 0.318 | 0.152 | 2.59 (0.82) | 0.231 | 0.360 | 2.09 (0.81) | 0.543 | 0.413 |
| UrT4 | 2.17 (0.81) | 0.491 | 0.402 | 2.23 (0.82) | 0.409 | 0.322 | 2.10 (0.81) | 0.605 | 0.587 |
| UnT1 | 2.36 (0.88) | 0.330 | -0.074 | 2.44 (0.92) | 0.356 | -0.057 | 2.26 (0.81) | 0.184 | -0.410 |
| UnT2 | 2.64 (1.03) | 0.443 | -0.163 | 2.75 (1.06) | 0.350 | -0.327 | 2.50 (0.97) | 0.532 | 0.107 |
| UnT3 | 2.70 (0.96) | 0.123 | -0.190 | 2.76 (0.95) | 0.127 | -0.151 | 2.62 (0.97) | 0.131 | -0.240 |
| UnT4^a^ | 2.43 (0.95) | 0.393 | -0.030 | 2.30 (0.93) | 0.457 | 0.039 | 2.59 (0.95) | 0.319 | -0.047 |
| Note. Value range is 1-5 for all items.  ^a^ Construction and IT workers reported higher value compared to HSO on this particular item**.** | | | | | | | | | |

**Table S3.** Confirmatory factor analyses BITS, excluding the item UnT4 (“...if other people made less mistakes”). Fit indices.

| Model | χ2(df)* | CFI | RMSEA | SRMR |
| --- | --- | --- | --- | --- |
| 2-factors solution, total sample (N=1,716) | 63.4 (13) | .985 | .048 (.036-.060) | .022 |
| 2-factors solution, human service workers (n=966) | 45.0 (13) | .983 | .050 (.035-.067) | .025 |
| 2-factors solution, non-‘human service’ workers (n=750) | 38.1 (13) | .983 | .051 (.032-.070) | .027 |

* with estimator MLM

**Table S4.** Fit indices for measurement invariance testing in Human service workers (Nurses vs Teachers), excluding the

item UnT4 (“...if other people made less mistakes”).

| Model | χ2 (df) | CFI | RMSEA (90% CI) | SRMR | Model comp | Δχ2 (Δdf)^b^ | ΔCFI | ΔRMSEA | ΔSRMR | |
| --- | --- | --- | --- | --- | --- | --- | --- | --- | --- | --- |
| M1: Configural Invariance | 60.0 (26) | .982 | .052 (.035-.069) | .029 | -- | -- | -- | -- | -- | |
| M2: Metric Invariance | 68.0 (31) | .981 | .050 (.034-.066) | .037 | M2 vs M1 | 8.0(5) | .001 | -.002 | .008 | |
| M3: Scalar Invariance | 91.8 (36) | .971 | .057 (.042-.071) | .042 | M3 vs M2 | 25.3***(5) | .010 | .007 | .005 | |
| Note. *N* = 966; Nurses, *n* = 464; Teachers; *n* = 502.  *** *p* < .001.  ^a^ with MLM correction | | | | | | | | | |  |

**Table S5.** Fit indices for measurement invariance testing in Non-‘human service’ workers (Construction vs IT workers), excluding the

item UnT4 (“...if other people made less mistakes”).

| Model | χ2 (df) | CFI | RMSEA (90% CI) | SRMR | Model comp | Δχ2 (Δdf)^b^ | ΔCFI | ΔRMSEA | ΔSRMR | |
| --- | --- | --- | --- | --- | --- | --- | --- | --- | --- | --- |
| M1: Configural Invariance | 46.9 (26) | .986 | .046 (.024-.067) | .031 | -- | -- | -- | -- | -- | |
| M2: Metric Invariance | 54.7 (31) | .984 | .045 (.024-.064) | .039 | M2 vs M1 | 7.7 (5) | .002 | -.001 | .008 | |
| M3: Scalar Invariance | 81.2 (36) | .969 | .058 (.041-.075) | .046 | M3 vs M2 | 29.2***(5) | .013 | .013 | .007 | |
| Note. *N* = 750; Construction workers, *n* = 324; IT workers, *n* = 426.  *** *p* < .001.  ^b^ with MLM correction | | | | | | | | | |  |

**Table S6.** Fit indices for measurement invariance testing between Human service workers and Non-‘human service’ workers, excluding

the item UnT4 (“...if other people made less mistakes”).

| Model | χ2 (df) | CFI | RMSEA (90% CI) | SRMR | Model comp | Δχ2 (Δdf)^b^ | ΔCFI | ΔRMSEA | ΔSRMR | |
| --- | --- | --- | --- | --- | --- | --- | --- | --- | --- | --- |
| M1: Configural Invariance | 83.0 (26) | .983 | .051 (.039-.063) | .026 | -- | -- | -- | -- | -- | |
| M2: Metric Invariance | 96.9 (31) | .980 | .050 (.039-.061) | .033 | M2 vs M1 | 13.9*(5) | .006 | -.001 | .007 | |
| M3: Scalar Invariance | 190.9(36) | .954 | .071 (.061-.081) | .047 | M3 vs M2 | 103.8***(5) | .026 | .021 | .014 | |
| M3a: Partial Scalar Invariance | 121.9(35) | .974 | .054 (.044-.064) | .036 | M3a vs M2 |  | .006 | .004 | .003 | |
| Note. *N* = 1,716; Human service workers (nurses and teachers), *n* = 966; Non-‘human service’ workers (construction and IT workers), *n* = 750.  *** *p* < .001.  ^a^ the constraints of equal intercepts for one item (UrT3: “…put you into an awkward position”) of the unreasonable tasks’ dimension was relaxed  ^b^ with MLM correction | | | | | | | | | |  |

**Fig. S1** Confirmatory factor analysis (CFA) solution with the two factors unreasonable and unnecessary tasks, excluding the item UnT4 ( “...if other people made less mistakes”). Standardized item factor loadings are presented for the total study sample followed in parentheses by stratified (by multigroup analysis) factor loadings for the two occupational categories (registered nurses and teachers; construction and IT workers).

UrT3

UrT1

UrT2

UrT4

UnT3

UnT1

UnT2

.66* (.67*; .63*)

.69 (.68; .69)

.67 (.63; .70)

.79 (.77; .79)

.75 (.75; .78)

.78 (.77; .76)

.60 (.59; .59)

.73 (.79; .65)

Note. Total study sample, *N =* 1,716 individuals; Human service workers sample, *n =* 966 individuals; Non-‘human service’ workers sample, *n =* 750 individuals.

No modification adding error-covariances between items included.

UrT is unreasonable tasks; UnT is unnecessary tasks

Job satisfaction

UrT3

UrT1

UrT2

UrT4

UnT3

UnT1

UnT2

UnT4

Burn3

Burn1

Burn2

Burn4

Burn5

Burn7

Burn6

.39* (.36*; .45*)

-.14* (-.15*; -.13*)

.07ns (.08ns; .04ns)

-.30* (-.29*; -.33*)

.66* (.68*; .65*)

-.34* (-.41*; -.23*)

**Fig. S2** Standardized associations: All groups (in parentheses; results from multigroup analyses: HSW (registered nurses & teachers); non-HSW (construction and IT workers). No modification adding error-covariances between items was used. Fit indices for total study sample χ2(df) = 332.35(85), CFI = .976, RMSEA = .042 (90% CI .037–.047) and SRMR = .027); for (partial scalar) multigroup analysis χ^2^(df) = 528.77 (191), CFI = .968, RMSEA = .046 (90% CI .041–.051) and SRMR = .040).

UrT is unreasonable tasks; UnT is unnecessary tasks
